# Characterization of hyperlipidemia secondary to mitotane in adrenocortical carcinoma

**DOI:** 10.1530/EO-21-0021

**Published:** 2022-02-07

**Authors:** Nadia Gagnon, Sophie Bernard, Martine Paquette, Catherine Alguire, André Lacroix, Pierre-Olivier Hétu, Harold J Olney, Isabelle Bourdeau

**Affiliations:** 1 Division of Endocrinology, Department of Medicine, Centre de Recherche du Centre hospitalier de l’Université de Montréal (CRCHUM), Montreal, Quebec, Canada; 2 Lipids, Nutrition and Cardiovascular Prevention Clinic of the Montreal Clinical Research Institute, Montreal, Québec, Canada; 3 Department of Biochemistry, Centre hospitalier de l’Université de Montréal (CHUM), Montreal, Quebec, Canada; 4 Department of Medicine, Centre hospitalier de l'Université de Montréal (CHUM), Montreal, Quebec, Canada

**Keywords:** adrenocortical carcinoma, hypercholesterolemia, hyperlipidemia, mitotane

## Abstract

**Background:**

This study examined the magnitude of changes and the time required to observe maximal changes in LDL-c, HDL-c, triglycerides (Tg) and non-HDL-c after the introduction of mitotane.

**Methods:**

Retrospective study of 45 patients with adrenocortical carcinoma who were treated at the Centre hospitalier de l’Université de Montréal. Clinical and biochemical data were collected, including lipid profiles before and during the first year of treatment with mitotane.

**Results:**

Among the 45 studied patients, 26 (58%) had a complete lipid profile before the introduction of mitotane and at least 1 lipid profile during the first year of treatment, and 19 patients (42%) had a lipid profile following initiation of the treatment. Among the 26 patients who had lipid profiles before and after the introduction of mitotane, the increase of LDL-c was 2.19 mmol/L (76%) (*P*< 0.0001), HDL-c was 0.54 mmol/L (35%) (*P*= 0.0002), Tg was 1.80 mmol/L (129%) (*P*< 0.0001) and non-HDL-c was 2.73 mmol/L (79%) (*P*< 0.0001). Between the first and the sixth month of mitotane treatment, peak values (*n*  = 45) of LDL-c and non-HDL-c were reached in 42 patients (93%) and 37 patients (82%), respectively, whereas peak values of HDL-c were reached after 6 months of mitotane treatment in 29 patients (66%). The peak value of Tg was almost equal throughout the first year. The mean peak values of HDL-c, Tg and non-HDL-c showed significant associations with their respective mitotane concentrations (β = 0.352, *P*= 0.03; β = 0.406, *P*= 0.02 and β = 0.339, *P*= 0.05).

**Conclusion:**

The introduction of mitotane produces a clinically significant elevation of lipid parameters (LDL-c, HDL-c, Tg and non-HDL-c) during the first year of treatment.

## Introduction

Adrenocortical carcinoma (ACC) has an incidence of approximately 1–2 per million population per year (Bourdeau *et al.* 2013). Staging at presentation remains one of the key prognostic factors with an expected 5-year survival of 80% for patients with stage I and 13% for patients with stage IV (Fassnacht *et al.* 2009). Mitotane (o,p’DDD) is an adrenolytic drug and remains the most effective adjuvant agent after curative surgery as well as for palliative intent in stage IV ACC. Although its molecular mechanisms of action are not fully understood, mitotane is reported to induce endoplasmic reticulum (ER) stress, impairment of steroidogenesis and apoptosis in adrenocortical cells (Sbiera *et al.* 2015, Kroiss *et al.* 2016). Mitotane is a lipophilic agent that accumulates in circulating lipoprotein fractions and high-lipid-containing tissues, and dyslipidemia has been observed in mitotane-treated patients (Terzolo *et al.* 2000, Daffara *et al.* 2008, Shawa *et al.* 2013). Shawa *et al.* reported significant increases in total cholesterol (TC), contributed mainly by increasing LDL-c, HDL-c and triglycerides (Tg) in a cohort of 38 patients with ACC (Shawa *et al.* 2013). In a smaller cohort of 17 consecutive patients with ACC, Daffara *et al.* also reported a significant increase of TC and HDL-c, while Tg levels did not change significantly (Daffara *et al.* 2008). The HDL-c increase correlated with plasma mitotane concentrations in both studies (Daffara *et al.* 2008, Shawa *et al.* 2013).

Patients with ACC have a high risk of recurrence, but treatment with adjuvant mitotane may prolong recurrence-free survival in patients with radically resected ACC (Berruti *et al.* 2017). Actual therapeutic guidelines recommend at least 2 years of adjuvant treatment with mitotane at targeting therapeutic concentrations of 14–20 mg/L in patients with a high risk of recurrence (Fassnacht *et al.* 2018). Monitoring lipid profiles every 3–4 months is recommended after the introduction of mitotane (Fassnacht *et al.* 2018).

Currently, limited data are available on the degree of alterations of the lipid profile expected after the introduction of mitotane therapy, the time required to observe the peak of variation in lipid parameters and the evolution of cardiovascular health in these patients. The objective of this retrospective study was to determine the magnitude of lipids alterations (LDL-c, HDL-c, Tg and non-HDL-c) and the time needed to observe the maximal increase in lipids parameters after the introduction of mitotane in patients with ACC.

## Materials and methods

We reviewed the charts of consecutive patients with ACC referred and followed at the *Centre hospitalier de l’Université de Montréal* (*CHUM*), a Canadian academic referral center for adrenal tumors. Clinical data were collected, including age, sex, past medical history of dyslipidemia and its treatment, tumor hormonal secretion, tumor histology, *de novo* dyslipidemia and/or lipid-lowering drugs initiated after the introduction of mitotane. All cardiovascular events following the introduction of treatment were collected.

Biochemical data were collected, including 12-h fasting lipid profiles before the introduction of mitotane, 1 month after and every 3 months during the first year of treatment. The peaks of LDL-c, HDL-c, Tg and non-HDL-c levels were defined as the highest value found during the first year of treatment. Presented values of LDL-c were all calculated as no directly measured values were obtained. Plasma mitotane concentration values were also collected during the first year. Plasma mitotane levels were assayed by HPLC. All mitotane concentrations were analyzed at the CHUM biochemistry laboratory. Lipid profiles were either analyzed at the CHUM biochemistry laboratory or at other accredited hospital laboratories in the province of Quebec. The ethics committee of the CHUM approved this study.

Descriptive statistics were used to summarize patient’s clinical characteristics. The average changes in lipids from baseline to peak concentration were assessed with a paired *t*-test. Only patients with an available lipid profile before and at least once after the introduction of the drug were included in the analysis. The relationship between mean peak LDL-c, HDL-c, Tg, non-HDL-c and corresponding plasma mitotane concentrations was assessed with a simple linear regression calculation. The relationship between mean peak lipid levels and corresponding mitotane concentrations was also analyzed by an ANOVA test according to mitotane subgroups: non-therapeutic (<14 mg/L), therapeutic (between 14 and 20 mg/L) and toxic levels (>20 mg/L) of mitotane. A *P*-value less than 0.05 was used to assess statistical significance. All patients with at least one lipid profile following the introduction of mitotane were included in the analysis. Finally, to evaluate the strength of the linear relationship between clinical characteristics (age, sex and hypercorticism) and peak values of LDL-c, HDL-c or Tg, a Pearson’s correlation coefficient was used.

## Results

**Figure 1:**
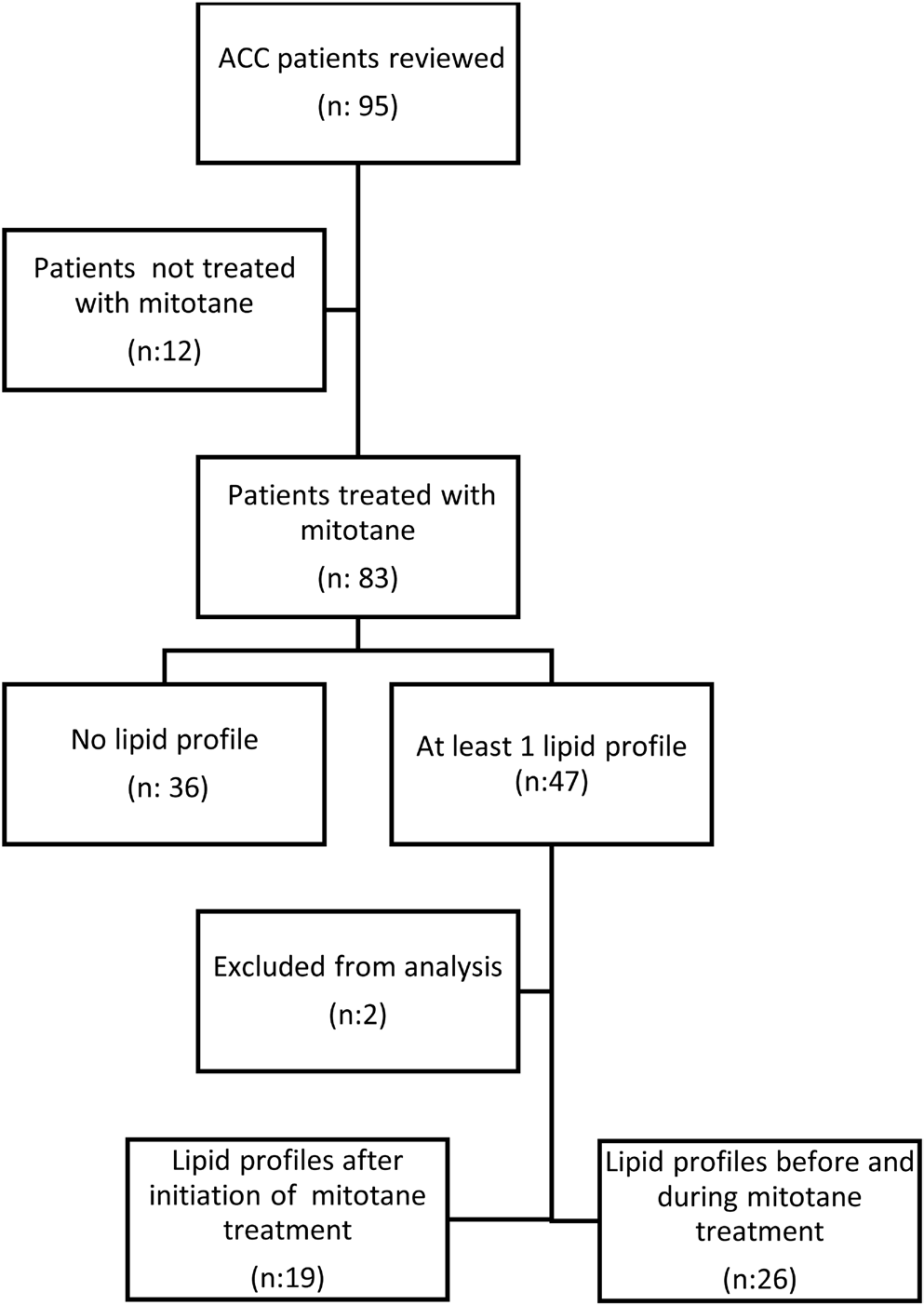
Summary of selection of the study cohort.

We reviewed the charts of 95 consecutive patients with ACC referred and followed at our center. Summary of selection for the study cohort is presented in Fig. 1. One patient had only a lipid profile before the introduction of mitotane without follow-up profiles and another patient had a very elevated Tg level at baseline, representing an extreme outlier or another underlying pathology. They were both excluded from the analysis. A total of 45 informative patients were included in the study cohort. Among the patients with complete lipid profiles, nine patients (34.6%) had lipid profiles at 1, 3, 6, 9 and 12 months.

A summary of the clinical characteristics of the entire cohort is presented in Table 1. Chemotherapy was given to 20 patients: 4 patients with stage III and 16 patients with stage IV ACC. Mitotane was given alone as adjuvant treatment in 25 patients (55%). Before the introduction of mitotane, 10 patients (22%) had a past medical history of treated dyslipidemia. Also, 6 patients were current smokers, 11 patients were treated for high blood pressure, 3 patients had type 2 diabetes, 4 patients had a past medical history of coronary vascular disease and 6 patients had peripheral vascular disease.

**Table 1:** Clinical characteristics of the entire cohort of patients with ACC (*n*  = 45) and patient subgroup with a complete lipid profile before and after the introduction of mitotane (*n*  = 26). Data for continuous variables are expressed as median ± s.d. Categorical variables are expressed as frequency (*n* (%)).

Variables	*n*= 26	*n*= 45
Age (years)	50 ± 13	50 ± 14
Sex		
Male	6 (23%)	10 (22%)
Female	20 (77%)	35 (78%)
ENSAT stage		
I	0 (0%)	2 (4%)
II	9 (35%)	11 (24%)
III	11 (42%)	15 (33%)
IV	6 (23%)	17 (38%)
Median tumor diameter (cm)	11,58 (2.5–21.5)	11,38 (2.5–24)
Median Weiss score	6 (*n* = 16) (3–9)	6 (*n* = 26) (3–9)
Functional status		
Secreting tumors	16 (62%)	29 (64%)
Glucocorticoids only Aldosterone only Androgens only Glucocorticoids and androgens	5 3 2 6	10 4 2 13
Non-secreting tumors	3 (11%)	5 (11%)
Unknown	7 (27%)	11 (25%)
Concomitant treatments		
Cytotoxic chemotherapy	9 (35%)	20 (44%)
Past medical history of treated dyslipidemia		
Yes	6 (23%)	10 (22%)
No	20 (77%)	34 (76%)

### Changes in LDL-c, HDL-c, Tg and non-HDL-c during the first year of mitotane treatment

**Figure 2:**
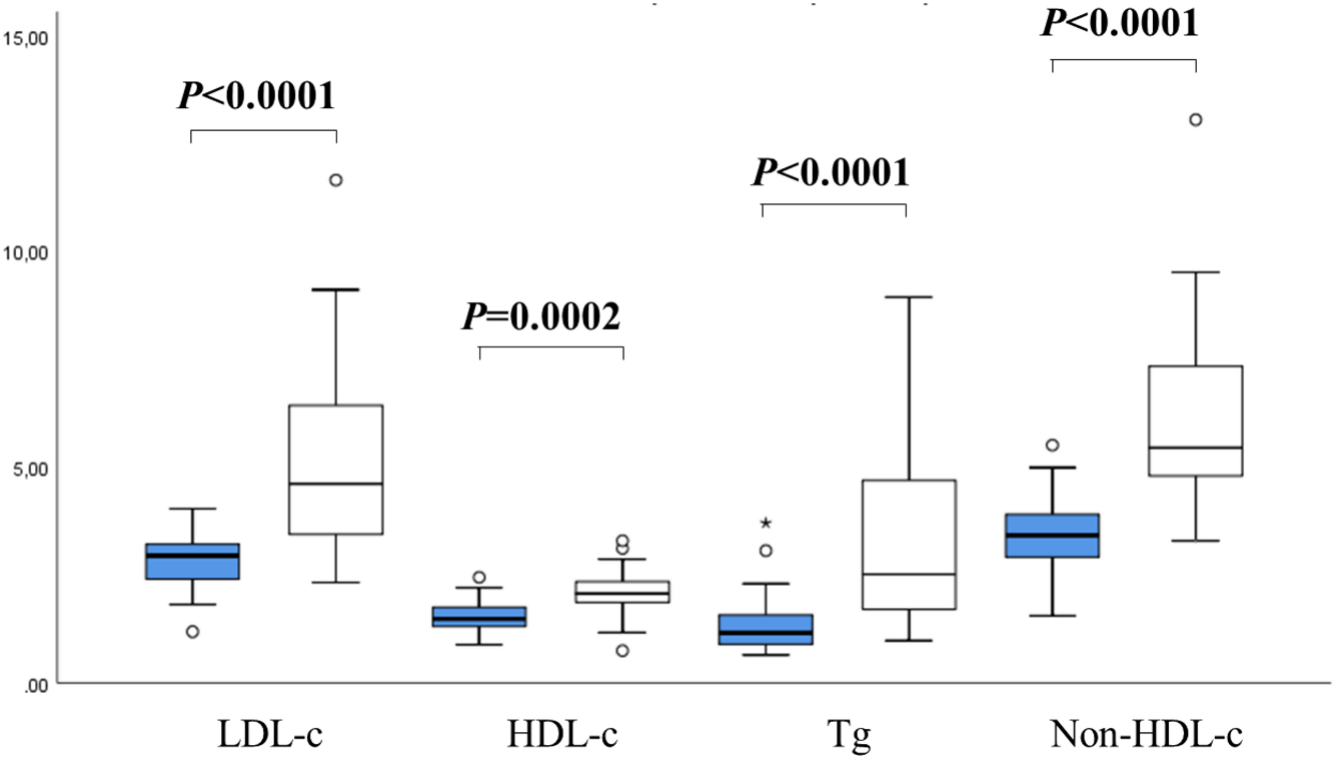
Basal and peak values of LDL-c, HDL-c, Tg and non-HDL-c during the first year of treatment with mitotane of 26 patients with ACC. Tg, triglycerides. *P*-values for paired *t*-test between basal and peak lipid values. Blue rectangles represent baseline values, and white rectangles represent peak values. HDL-c, high density lipoprotein cholesterol; LDL-c, low density lipoprotein cholesterol; Tg, triglycerides.

**Figure 3:**
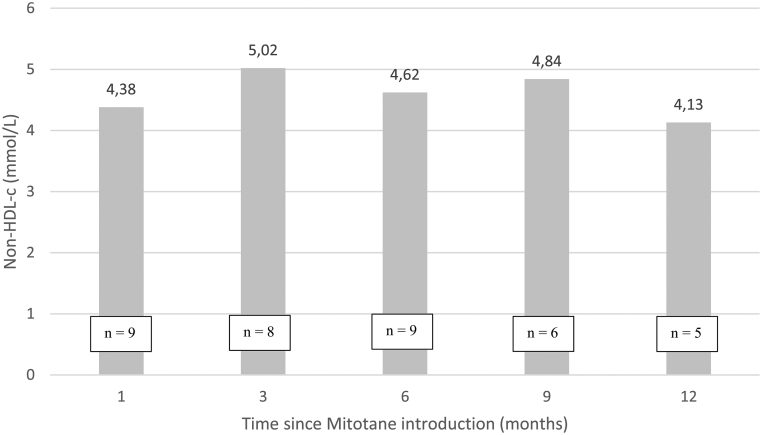
Evolution of non-HDL-c levels throughout 12 months of mitotane treatment. Data are presented as the mean. HDL-c, high density lipoprotein cholesterol.

Among the patients who had complete lipid profiles before and after the introduction of mitotane (*n*  = 26), the mean baseline values of LDL-c, HDL-c, Tg and non-HDL-c were respectively 2.88 mmol/L (±0.67), 1.54 mmol/L (±0.37), 1.39 mmol/L (±0.74) and 3.44 mmol/L (±0.87). The mean peak values of LDL-c, HDL-c, Tg and non-HDL-c during the first year of treatment were respectively 5.07 mmol/L (±2.20), 2.08 mmol/L (±0.57), 3.19 mmol/L (±2.07) and 6.17 mmol/L (±2.26). There was a mean elevation of 2.19 mmol/L (76%) in LDL-c (*P* < 0.0001), 0.54 mmol/L (35%) in HDL-c (*P* = 0.0002), 1.80 (129%) in Tg levels (*P*< 0.0001) and 2.73 mmol/L (79%) in non-HDL-c (*P*< 0.0001). These results are presented in Fig. 2. Results of non-HDL-c during the year of treatment are presented in Fig. 3.

Patients with a past medical history of treated dyslipidemia (*n*  = 6) showed a smaller LDL-c increase of 2.78 mmol/L (±0.85) compared to 4.50 mmol/L (±2.48) (1.72 mmol/L, 69%) in the rest of the cohort, a smaller HDL-c increase of 1.71 mmol/L (±0.27) compared to 2.21 mmol/L (±0.59) (0.50 mmol/L, 30%) and a more important Tg increase of 1.28 mmol/L (±0.65) compared to 3.57 mmol/L (±3.08) (2.29 mmol/L, 228%). The initial lipid treatment was niacin (n:1), fluvastatin (n:1), atorvastatin (n:2) and rosuvastatin (n:2). After the introduction of mitotane, fluvastatin was modified for atorvastatin in one case and atorvastatin was stopped for unknown reasons in one other patient. All the other treatments remained the same. During the first year of mitotane treatment, nine patients initiated lipid therapy: atorvastatin in four cases, rosuvastatin in four cases and one case of a combination of atorvastatin with ezetimibe.

### Time required to observe the peak values in lipid parameters during the first year of mitotane treatment

**Figure 4:**
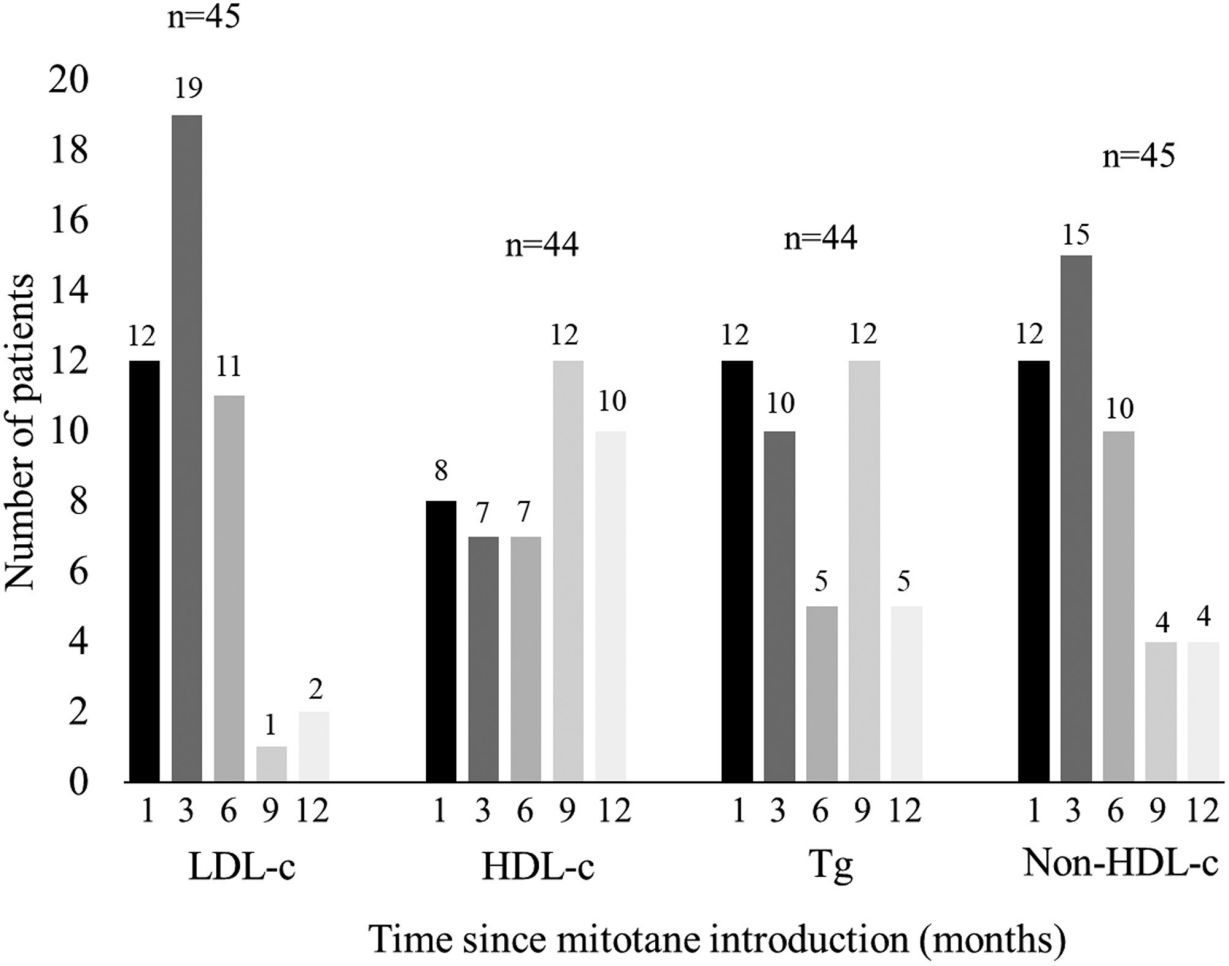
Timing of peak values for LDL-c, HDL-c, Tg and non-HDL-c from mitotane introduction. HDL-c, high density lipoprotein cholesterol; LDL-c, low density lipoprotein cholesterol; Tg, triglycerides.

Of the entire cohort with lipid profiles after the introduction of mitotane (*n*  = 45), the peak values of LDL-c were observed between the first and sixth month of treatment for 42/45 patients (93%). The peak values of HDL-c were observed after 6 months of treatment for 29/45 patients (66%). The elevation of Tg up to peak values was almost equal during the first year of treatment. The peak values of non-HDL-c were observed between the first and sixth months for 37/45 patients (82%). These results are presented in Fig. 4.

### Association between LDL-c, HDL-c, Tg and non-HDL-c peak values and plasma mitotane concentration

We observed that the mean peak value of LDL-c was 4.72 mmol/L when the corresponding mitotane level was non-therapeutic (<14 mg/L), 4.97 mmol/L at therapeutic levels (between 14 and 20 mg/L) and 5.36 mmol/L when reaching a toxic level (>20 mg/L) (*P*  = 0.93). The mean peak value of HDL-c was 1.88 mmol/L when the corresponding mitotane level was non-therapeutic, 2.72 mmol/L when therapeutic and 2.33 mmol/L when reaching a toxic level (*P*  = 0.02). The mean peak value of Tg was 2.46 mmol/L when the corresponding mitotane level was non-therapeutic, 3.22 mmol/L when therapeutic and 3.64 mmol/L when reaching a toxic level (*P*  = 0.16). The mean peak value of non-HDL-c was 5.55 mmol/L when the corresponding mitotane level was non-therapeutic, 6.64 mmol/L when therapeutic and 6.86 mmol/L when reaching a toxic level (*P*  = 0.48). The mean peak values of LDL-c showed no correlation with mitotane concentration (β = 0.196, *P*= 0.25). There was a positive significant correlation between the mean peak values of HDL-c, Tg and non-HDL-c and their respective mitotane concentrations (β = 0.352, *P*= 0.03; β = 0.406, *P*= 0.02 and β = 0.339, *P*= 0.05, respectively). Interestingly, if patients previously treated with lipid-lowering agents were removed from the analysis, there was still no association between mean peak values of LDL-c and mitotane concentration.

### Predictors of lipid change with mitotane treatment

No significant correlations were found either between the change in LDL-c, HDL-c or Tg and the age at diagnosis (LDL: *P*= 0.61, HDL: *P*= 0.69 and Tg: *P*= 0.75) or with gender (LDL-c: *P*= 0.93, HDL-c: *P*= 0.70 and Tg: *P*= 0.62). There was also no significant correlation between change in LDL-c, HDL-c and Tg and preoperative hypercorticism (LDL-c: *P*= 0.65, HDL-c: *P*= 0.29, Tg: *P*= 0.49). Among the patients (*n*  = 12/26) who presented hypercorticism before surgery, the mean baseline values of LDL-c, HDL-c and Tg were respectively 2.84 mmol/L (±0.75), 1.62 mmol/L (±0.43) and 1.42 mmol/L (±0.95). The mean peak values of LDL-c, HDL-c and Tg during the first year of treatment were 4.59 mmol/L (±1.94), 1.99 mmol/L (±0.59) and 3.37 mmol/L (±1.74). The respective increases were 1.75 mmol/L (67%) for LDL-c, 0.38 mmol/L (32%) for HDL-c and 1.94 mmol/L (176%) for Tg. After surgery, no patients remained in a residual hypercorticism state as tumors were completely resected and hydrocortisone replacements were well adjusted.

### Cardiovascular events during the first year of mitotane treatment

No new cardiovascular events were retrospectively identified during the first year of mitotane treatment, including in patients with a past medical history of coronary or peripheral vascular disease (*n*  = 10).

## Discussion

In our cohort of patients with stage I–IV ACC, mitotane treatment was associated with a clinically significant increase in LDL-c, HDL-c, Tg and non-HDL-c during the first year. In a subgroup of patients, we analyzed lipid profiles collected every 3 months throughout the year. To our knowledge, our study is the first to report that LDL-c and non-HDL-c levels increased in the first 6 months of treatment, while HDL-c mainly increased after 6 months and Tg elevation was almost equally distributed throughout the first year of mitotane treatment. The relative increase was more substantial for LDL-c (76%), Tg (129%) and non-HDL-c (79%) than for HDL-c (35%). This is concordant with previous studies that reported an increase in TC and HDL-c (Maher *et al.* 1992, Terzolo *et al.* 2000, Allolio *et al.* 2004, Daffara *et al.* 2008). Shawa *et al.* reported that their cohort of ACC patients showed an increase of LDL-c but mainly of HDL-c (Shawa *et al.* 2013). More recently in 2020, Basile *et al.* reported lipid profiles of 74 ACC patients receiving mitotane. They observed similar results to our study over a period of 6 months but found no significant changes in LDL-c (Basile *et al.* 2020). In 2021, Vikner *et al.* also found a significant increase in TC, LDL-c, HDL-c and Tg in a cohort of 50 ACC cases treated with mitotane over a period of 6 months (Vikner *et al.* 2021).

The mechanism of TC, LDL-c and HDL-c increase under mitotane treatment is still unclear. It was suggested that mitotane increases cholesterol synthesis by its ability to block cytochrome P450-mediated reactions, impairing the formation of oxysterols which are responsible for hepatic cholesterol synthesis downregulation. Recently, mitotane was shown to confer adrenal-specific cytotoxicity and downregulate steroidogenesis by inhibition of sterol-O-acyl-transferase 1 (SOAT1) leading to lipid-induced ER stress (Sbiera *et al.* 2015). Mitotane absorption also involves chylomicron binding, and mitotane binds to lipid membranes, modifying its physico-chemical properties depending on the membrane lipid composition (Scheidt *et al.* 2016).

The majority of mitotane is bound to lipoproteins in serum (Hescot *et al.* 2015, Kroiss *et al.* 2016). Kroiss *et al.* found in the serum of 14 ACC patients treated with mitotane that only 12.3 ± 5.5% was lipoprotein-free mitotane. They also found that lipoprotein-free mitotane correlated with serum mitotane concentrations and inversely with TC and Tg concentrations (Kroiss *et al.* 2016). They confirmed the previous suggestion that lipoprotein-free mitotane is the active form of the drug and that lipoproteins decrease the bioavailability of active mitotane (Hescot *et al.* 2015, Kroiss *et al.* 2016). This certainly has potential clinical repercussions. In our cohort, even if the sample was small (*n*  = 6/26), patients who were treated for dyslipidemia before the introduction of mitotane showed a smaller increase in LDL-c and HDL-c levels and a more important Tg increase. It was previously suggested that mitotane-induced dyslipidemia may interfere with the tumor activity of the drug (Hescot *et al.* 2015). Hescot *et al.* reported a greater efficacy of lipoprotein-free mitotane and analyzed retrospectively a cohort of 26 ACC in whom statin therapy was associated with a higher rate of tumor control (Hescot *et al.* 2015). In contrast, Sbiera *et al.* reported that at low mitotane concentrations, atorvastatin antagonized the cytotoxic effect of mitotane on adrenal cortex cells by decreasing the amount of free cholesterol responsive to lipotoxic ER stress (Sbiera *et al.* 2015). However, Weigand *et al.*, demonstrated that SOAT1 expression was not correlated with clinical recurrence-free survival and disease-specific survival in ACC patients with mitotane monotherapy (Weigand *et al.* 2020).

For a subgroup of patients, we collected lipid profiles every 3 months for the first year and were able to better estimate the timing of increases in LDL-c, HDL-c, Tg and non-HDL-c after the introduction of mitotane. We observed the increase in LDL-c and non-HDL-c for most patients (93 and 82%) during the first 6 months of mitotane treatment. For 2/3 of the patients (66%), the increase in HDL-c was mainly after 6 months of treatment. This is consistent with the Shawa *et al.* study that reported a median time for HDL-c peak of 8 months (234.5 days, 31–1426 days) (Shawa *et al.* 2013). There was no precise timing for the maximum increase in Tg as it was almost equal during the first year.

We observed a significant positive correlation between the mean peak values of HDL-c, Tg and non-HDL-c and their respective mitotane concentrations. However, we did not observe any association between LDL-c and mitotane concentration. Although statin treatment has the main effect of lowering LDL-c compared to HDL-c and Tg, there was still no association between mean peak values of LDL-c and mitotane concentration when patients previously treated with lipid-lowering agents were removed from the analysis. This result is possibly explained by the small number of patients. The association between mean peak values of HDL-c and mitotane concentrations was previously described in two studies (Daffara *et al.* 2008, Shawa *et al.* 2013). Paci *et al.* reported the case of a 39-year-old male without a past medical history of dyslipidemia and with stage IV ACC treated with mitotane, in whom several mitotane levels were >30 mg/L with excellent neurological tolerance. Based on this case, the authors suggested an overestimation of mitotane levels in hypercholesterolemic and hypertriglyceridemic patients compared to normolipidemic patients. A matrix effect would result in an overestimation of o,p’DDD in the presence of hyperlipidemia and the degree of this overestimation would positively correlate with the severity of hyperlipidemia. Such interference could be corrected by diluting plasma samples or by using phospholipid removal cartridges (Paci *et al.* 2016). This highlights the clinical importance of completing a concomitant lipid profile when obtaining a mitotane level. Glucocorticoids excess may lead to an increase in TC, LDL-c and Tg levels and a decrease in HDL-c, but we did not observe any correlation between any lipid increase and the presence of preoperative hypercorticism (Greenman *et al.* 2010).

It remains unclear if the increase in LDL-c has an impact on the cardiovascular evolution of patients. We did not observe any new events during the first year of mitotane treatment. However, this is certainly too short of a time period to evaluate cardiovascular events from new-onset dyslipidemia. In addition, a much larger number of patients studies long-term would be required to identify cardiovascular outcome effects. To our knowledge, only one case was previously described with a cardiovascular event related to mitotane introduction: a 58-year-old man without a past medical history of dyslipidemia or cardiovascular disease who developed new angina requiring revascularization using a percutaneous coronary intervention after starting a low dose of mitotane (Tada *et al.* 2014).

The design of our retrospective study presents some limitations. Of the entire cohort, 43% did not have any of our now standard 3-monthly lipid profile sampling during their mitotane treatment. This is explained by the recognition of the phenomenon and development of local therapeutic consensus recommendations since 1995. Actual therapeutic guidelines recommend a complete lipid profile every 3–4 months and if LDL-c and HDL-c are consistently increased, the recommendation is to consider treatment with statins not metabolized by CYP 3A4 (Fassnacht *et al.* 2018). The peaks of LDL-c, HDL-c, Tg and non-HDL-c levels were defined as the highest value found during the first year of treatment. In the presence of new-onset dyslipidemia, a lipid-lowering treatment might have been started during this first year, influencing the peak values that were reached. As mentioned previously, a lipid profile is usually prescribed with a fasting period of 12 h at our center, but it was not possible to confirm that each profile was collected in the fasting state, which could have impacted measured Tg levels. Despite these limitations, we were able to evaluate complete lipid profiles drawn every 3 months for nine ACC patients during the first year of mitotane treatment, albeit with more success in our more recent referrals.

Given that lipoprotein-free mitotane is the active form of the drug and that lipoproteins decrease the bioavailability of active mitotane (Hescot *et al.* 2015, Kroiss *et al.* 2016), more prospective studies are needed to confirm whether statin therapy is beneficial for patients with ACC and should examine not only the cardiovascular risk but also the antitumoral effects of mitotane.

In conclusion, we report data on a cohort of consecutive patients with ACC and analyzed their lipid profiles, which confirmed clinically significant increases in LDL-c, HDL-c, Tg and non-HDL-c during the first year of mitotane treatment. This is the first report to describe that LDL-c and non-HDL-c increased in the first 6 months of treatment with mitotane, while HDL-c mainly increased after 6 months and Tg elevation was almost equal throughout the first year. The impact on the cardiovascular health of these patients remains to be determined. Improved patient care should result from a better understanding of the impact of lipoprotein-free mitotane, the co-administration of lipid-lowering drugs and their effects on the outcome of these patients. With this perspective, future studies should explore whether mitotane treatment has the same tumoral efficacy in normolipidemic vs hyperlipidemic patients.

## Declaration of interest

The authors declare that there is no conflict of interest that could be perceived as prejudicing the impartiality of the research reported.

## Funding

This study was supported in part by a salary grant to IB from Fonds de Recherche du Québec-Santéhttp://dx.doi.org/10.13039/501100000156 (FRQ-S).

## Author contribution statement

N G, S B and I B designed the study. N G and C A collected the data. N G, S B, M P and I B contributed to the interpretation of data and discussion. I B, A L, H O were involved in clinical management of the patients and P O H was responsible for mitotane level measurements. All authors have read, revised, and approved the manuscript.
